# Preparation of Alkali Activated Cementitious Material by Upgraded Fly Ash from MSW Incineration

**DOI:** 10.3390/ijerph192013666

**Published:** 2022-10-21

**Authors:** Hongwei Chen, Runbo Zhao, Wu Zuo, Guanghui Dong, Dongyang He, Tengfei Zheng, Changqi Liu, Hao Xie, Xinye Wang

**Affiliations:** 1 Jiangsu Provincial Key Laboratory of Materials Cycling and Pollution Control, School of Energy and Mechanical Engineering, Nanjing Normal University, Nanjing 210042, China; 2 Jiangsu Environmental Engineering Technology Co., Ltd., Nanjing 210019, China; 3 Zhenjiang Institute for Innovation and Development, Nanjing Normal University, Zhenjiang 212016, China

**Keywords:** municipal solid waste incineration fly ash, dioxin, chloride, calcium, alkali activated cementitious material

## Abstract

Utilization of municipal solid waste incineration fly ash (MSWI-FA) can avoid land occupation and environmental risks of landfill. In this paper, MSWI-FA was used to prepare alkali activated cementitious materials (AACMs) after two-step pretreatment. The ash calcination at 450 °C removed 93% of dioxins. The alkali washing with 0.2 g NaOH/g ash removed 89% of chlorine and retained almost 100% of calcium. The initial setting time of AACMs was too short to detect for 20% of MSWI-FA addition, and the prepared block had extensive cracks and expansion for CaClOH and CaSO_4_ inside. Alkaline washing pretreatment increased the initial setting time by longer than 3 min with 30% ash addition and eliminated the cracks and expansion. The significance of the factors for compressive strength followed the modulus of alkali activator > silica fume amount > alkaline washing MSWI fly ash (AW-MSWI-FA) amount. When the activator modulus was 1.2, 1.4 and 1.6, the blocks with 30% of AW-MSWI-FA had a compressive strength of up to 36.73, 32.61 and 16.06 MPa, meeting MU15 grade. The leaching test shows that these AACM blocks were not hazardous waste and almost no Zn, Cu, Cd, Pb, Ba, Ni, Be and Ag were released in the leaching solution.

## 1. Introduction

In cities with a dense population, there is generally a large production of municipal solid waste (MSW) and limited land for landfill. Incineration has the advantages of fast reduction volume (90%) and mass (70%), bacteria and virus elimination and energy production, so that it becomes the most important way to dispose of MSW for these cities [1]. However, the fly ash collected by the air pollutant control devices (APCDs) contains dioxins and heavy metals with relatively high concentrations, resulting in its classification as hazardous waste in many countries [2]. 

The main deposal method of municipal solid waste incineration (MSWI) fly ash is stabilization by chelator and solidification by cement, and then landfill [3]. Generally, the chelation effect is tested only before landfilling, regardless of whether it is still effective after landfilling. The difficulties in sampling and responsibility division are the main reasons for the lack of long-term stability supervision. In fact, it has been reported that the decomposition of lead (Pb) chelates in the six-year-old MSWI fly ash led to the release of Pb via the inorganic complex [4]. In recent years, the technology of collaborative disposal of MSWI fly ash in a cement kiln has sprung up, especially in China. In this technology, the low chlorine ash is directly fed into the cement kiln, while the high chlorine ash is washed by water for chlorine removal first before feeding [5]. However, there is no pre-treatment process for heavy metals removal, because it is considered that heavy metals are solidified inside the cement without environmental risk [6]. Actually, it is difficult to test the solidification effect because the feed ashes are much less than raw meal, so that the dilution effect also causes no change in the heavy metal leaching. In addition, the long-term stability of heavy metals in cement is still in question. Therefore, both the landfill and the resource utilization come with great environmental risks for heavy metals.

In the past decades, alkali-activated cementitious materials (AACMs) have been accepted as a family of effective and promising alternatives to Portland cement-based materials (PCMs) for their better physical and durability properties [7,8]. In addition, AACMs show greater performance on heavy metal ions’ immobilization than PCMs. Therefore, there is much research on MSWI fly ash solidification by AACMs for a long-term stability in landfill [9,10,11]. Furthermore, much of this research tried to use MSWI fly ash as one of the raw materials to produce AACMs (usually C–S–H hydration product) for the replacement of PCMs [12,13,14]. Compared with the traditional cement kiln collaborative disposal, the preparation of AACMs eliminates cement kiln, which in many cities is not available or is being shut down for its high carbon intensity and high pollution. Although reuse for AACMs preparation seems to be more environmentally friendly, there are still several issues, such as dioxins pollution, which cannot be reduced but only diluted in AACMs [13], high content chlorides, which corrode the steel reinforcement [15] and reduce the compressive strength of AACMs [16], et al.

**Figure 1:**
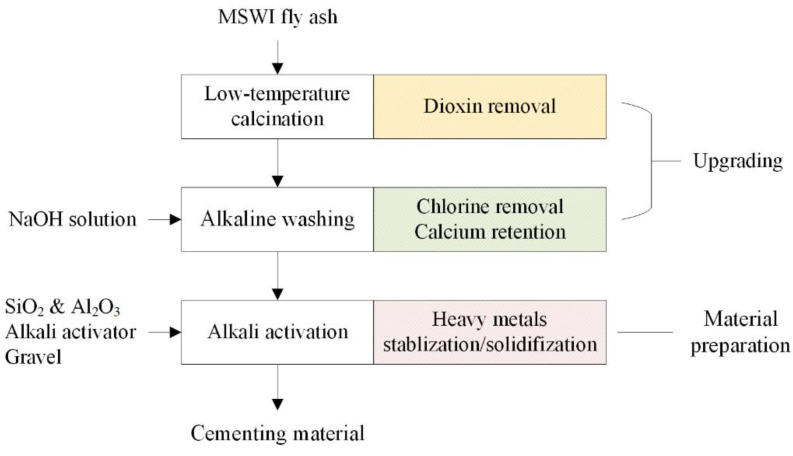
The process of MSWI fly ash upgrading and alkali activated material preparation.

According to the description above, in this paper, an innovative process was proposed to use MSWI fly ash to prepare AACMs, as shown in Figure 1. Firstly, low-temperature calcination and alkaline washing were used as ash upgrading, aiming at dioxins removal and chlorine removal. As opposed to traditional water washing, alkaline washing, or washing by NaOH solution, can not only dissolve NaCl and KCl but also precipitate calcium ion (Ca^2+^), realizing the dechlorination and the calcium retention at the same time. Then, the upgraded ash was mixed with other wastes rich in silicon (Si) and aluminum (Al), such as pulverized coal combustion (PCC) fly ash and silica fume (SF). Finally, alkali activation was carried out to prepare cementitious material. The effects of two upgrading steps and one material preparation step were investigated on the pollutants’ migration and performance of AACMs. The main difference between this paper and previous studies was the alkaline washing for ash pretreatment, which was proved effective on the setting time and compressive strength of AACMs.

## 2. Materials and Methods

### 2.1. Raw Materials

The MSWI fly ash was sampled from the ash silo of a MSWI plant, which is equipped with two mechanical grates with the incineration capacity of 2 × 350 t/d. The air pollution control process of this plant is selective non-catalytic reduction (SNCR) + semi-dry process (Ca(OH)_2_ slurry atomizing) + activated carbon injection (dioxin removal) + dry process (Ca(OH)_2_ powder injection) + bag filter. The PCC fly ash was sampled from the ash silo of a coal-fired power plant equipped with pulverized coal boiler with the power-generating capacity of 300 MW. The silica fume was derived from the ash of the industrial silicon smelting process with the SiO_2_ concentration of 97%.

**Figure 2:**
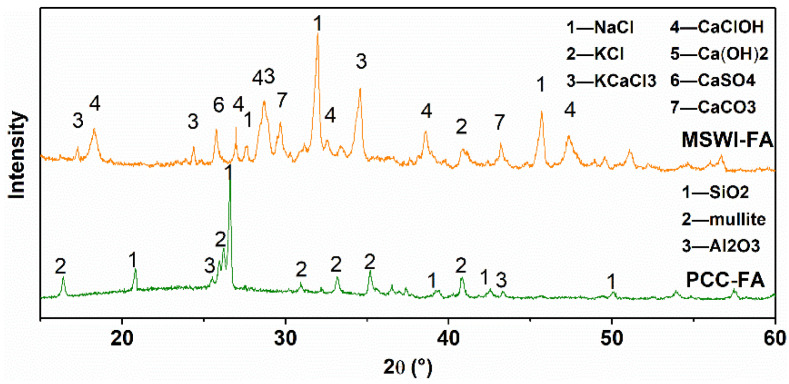
XRD patterns of MSWI fly ash and pulverized coal combustion fly ash.

The major components of two ashes are shown in Table 1 and Figure 2. The MSWI fly ash is composed of calcium, chlorine, sulfur, potassium and sodium in the chemical form of NaCl, KCl, KCaCl_3_, CaClOH, CaSO_4_ and CaCO_3_. The pulverized coal combustion fly ash is composed of silicon, aluminum and calcium in the chemical form of SiO_2_, Al_2_O_3_ and mullite.

### 2.2. Upgrading Methods

#### 2.2.1. Low-Temperature Calcination

Dioxins are a general term for polychlorinated dibenzo-p-dioxins (PCDDs) and polychlorinated dibenzofurans (PCDFs), which are in solid form at room temperature, almost insoluble in water [17]. Low-temperature heat treatment is an energy-saving and efficient method to remove dioxins, especially under a nitrogen atmosphere, preventing some precursor from reacting with oxygen and producing more dioxins in ash [18,19]. Song et al. [17] found that the dioxins’ removal efficiency was up to 98% after the calcination for 1 h at 450 °C under a nitrogen atmosphere. Therefore, the above condition was used for dioxins removal in this paper.

#### 2.2.2. Alkaline Washing

The MSWI fly ash after low-temperature calcination was mixed with the NaOH solution (0–0.075 mol/L) by magnetic stirring for 10 min following the liquid–solid ratio (L/S) of 1–12 mL/g. The slurry after stirring was filtered through the membrane with a pore size of 0.2 μm under the vacuum degree of −0.098 MPa. The filter residues that dried for 24 h at 105 °C were considered as the upgraded MSWI fly ash.

The calcium retention was achieved by the reaction of NaOH and Ca^2+^ following Equation (1). The Ca^2+^ dissolved in the washing slurry was in the form of CaCl_2_, mainly, and Ca(OH)_2_ and CaSO_4_, slightly [5,6]. Therefore, the NaOH solution converted the soluble CaCl_2_ into insoluble Ca(OH)_2_ so that all the calcium in the upgraded ash was in the form of Ca(OH)_2_ and CaSO_4_, which were in favor of AACMs preparation.

NaOH +0.5 Ca^2+^ = Ca(OH)_2_↓+ Na^+^ (1)

The chlorine removal fraction (*φ*_Cl_) and the calcium retention fraction (*ω*_Ca_) were calculated as Equations (2) and (3).

(2) $$\varphi_{\mathrm{Cl}}=\frac{C_{\mathrm{Cl}-\mathrm{filtrate}}\times V_{\mathrm{filtrate}}}{C_{\mathrm{Cl}-\mathrm{ash}}\times m_{\mathrm{ash}}}\times 100\%$$

(3) $$\omega_{\mathrm{Ca}}=(100-\frac{C_{\mathrm{Ca}-\mathrm{filtrate}}\times V_{\mathrm{filtrate}}}{C_{\mathrm{Ca}-\mathrm{ash}}\times m_{\mathrm{ash}}})\times 100\%$$

where *C*_Cl-filtrate_ and *C*_Ca-filtrate_ are the concentration of Cl^−^ and Ca^2+^ in the filtrate, respectively; *V*_filtrate_ and *m*_ash_ are the volume of filtrate of washing slurry and the mass of ash, respectively.

### 2.3. AACMs Preparation

#### 2.3.1. Preparation of Alkali Activator

The modulus (M) of the alkali activator was the ratio of the mole number of SiO_2_ and Na_2_O. The alkali activator with a different modulus (1.2, 1.4 and 1.6) was prepared by the mixture of NaOH, sodium silicate (modulus of 2.25, SiO_2_ content of 29.99%, Na_2_O content of 13.75%) and water. The alkali activator was uniformly prepared into an aqueous solution with a solid content of 40%, dissolved by ultrasonic shock and cooled for 24 h for later use. The amount of NaOH addition (G_2_) follows Equation (4).

(4) $$G_{2}=\frac{80{*G}_{1}*N*\left(M_{1}-M_{2}\right)}{62M_{2}*P}$$

where G_1_ is the mass of sodium silicate, N is its Na_2_O content, M_1_ is the initial sodium silicate modulus, M_2_ is the modulus of the preprepared base activator and P is the purity of sodium NaOH.

#### 2.3.2. Preparation Steps of AACMs

It was considered that the poly-sialate-siloxo (Si/Al = 2) configuration had better structural properties in the calcium-free gel system [20]. Furthermore, in the Ca(OH)_2_ containing system, silicon promotes the formation of hydrated C-S-H gel, C-A-S-H gel and N-S-A-H gel to increase the strength of AACMs [21]. Therefore, silica fume was added to increase the proportion of silicon in raw materials. During the preparation of AACMs, there were four raw materials of alkali activator, silica fume, pulverized coal combustion fly ash and alkaline washing MSWI fly ash (AW-MSWI fly ash), so that the orthogonal test was needed to determine the importance of the proportion of each raw material. Factors and levels of orthogonal test are shown in Table 2.

The arrangement of the orthogonal test was shown in Table 2. PSM1-PSM9 groups were a high calcium system. According to the cement’s optimum consistency (optimum fluidity of cement slurry), the water–cement ratio was chosen as 0.48 in all the experiments under the condition of the cement–sand ratio of 1:1 and the alkali activator addition amount following 8% Na_2_O of the total mass of powder.

The system without AW-MSWI fly ash was investigated to compare with the system with AW-MSWI fly ash. The preparation parameters of AACMs with pulverized coal combustion fly ash and silica fume are shown in Table 3. The water–cement ratio was chosen as 0.38 in all the experiments under the condition of the cement–sand ratio of 1:1 and the alkali activator addition amount following 8% Na_2_O of the total mass of powder.

Firstly, several steps included putting the powder with a different ratio into the stirring pot and mixing it for 20 min, then adding the pre-configured alkali activator and stirring quickly for 30 s. After that, it is injected into the 40 × 40 × 40 mm triple die and vibrating on the cement shaking table for 1 min to eliminate bubbles inside the slurry. Then, several steps included scraping off the excess part with a scraper, covering the plastic film, placing it at room temperature (20 ± 3 °C) for curing and placing it in the curing box with a temperature of 20 ± 3 °C and a humidity ≥ 95% for the expected test of the set age 24 h after removing the mold. The setting time of AACMs was investigated according to the test method of *Test methods for water requirement of normal consistency setting time and soundness of the portland cement* (GB/T1346-2011) [22]. The AACM blocks were cured for 3 d, 7 d and 28 d to test the compressive properties and heavy metal leaching toxicity. The strength test referred to the standard of *Fired common bricks* (GB/T 5101-2107) [23].

### 2.4. Testing and Characterization

An automatic potentiometric titrator (ZDJ-5B) was used for the chloride ion test. An electronic universal tensile testing machine (WDW-100 kN) was used to test the compressive strength of AACMs blocks. An inductively coupled plasma emission spectrometer (Plasma 3000) was used to analyze the metal ions concentration in solutions. The X-ray diffraction (Siemens-Bruker D5000) was carried out on a diffractometer with mono chromatized Cu-Kα radiation (λ = 1.5406 Å) at 2θ scanning range of 5–85° with the scanning speed of 5°/min. A scanning electron microscope (S-4800) was used to detect the surface morphology of the prepared AACMs.

## 3. Results and Discussion

### 3.1. Dioxins Removal by Low-Temperature Calcination

#### 3.1.1. Effect on Dioxins Removal

The dioxins distribution in the raw ash and the calcined ash is shown in Table 4. In MSWI fly ash, O8CDD accounted for 23.5% of dioxins but only 0.2% of toxicity equivalence quantity (TEQ) for its low toxic equivalency factor (TEF); 2,3,4,7,8- P5CDF accounted for 7.6% of dioxins but up to 39.6% of TEQ for its high TEF. After calcination, 93% of dioxins in total were removed effectively, and 2,3,4,7,8- P5CDF became the main resource of TEQ, accounting for as high as 55.3%. In general, the calcination had a high removal effect on all kinds of dioxins in ash, with the removal fractions between 88.1% and 99.9% by dechlorination and decomposition [24]. Specifically, the removal efficiency of dioxins was higher than that of furans because the ratio of PCDFs to PCDDs decreased from 0.74 to 0.22. It was reported that the longer calcination time, such as 3 h, can further improve the removal efficiency to 95% [17]. However, this insignificant increase in energy consumption by several times is definitely not cost-effective.

#### 3.1.2. Effect on Crystalline Phase Change

**Figure 3:**
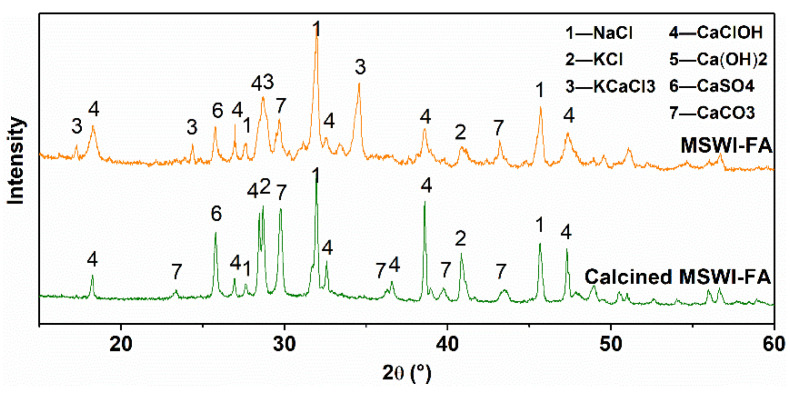
XRD patterns of MSWI fly ash and calcined ash.

The comparison of the crystalline phase of the raw ash and the calcined ash is shown in Figure 3. After the calcination for 1 h at 450 °C under an N_2_ atmosphere, the peaks of KCaCl_3_ disappeared completely and the peaks of KCl and CaClOH had higher intensity. Therefore, it is considered that KCaCl_3_ decomposed for the calcination to produce KCl and CaCl_2_. However, CaCl_2_ were found in Figure 3, so it either existed as the amorphous state or was converted to be CaClOH, which was considered to decompose above 500 °C higher than the calcination temperature [25,26]. In general, the low-temperature calcination did not change the main components of MSWI fly ash.

### 3.2. Chlorides Removal and Calcium Retention by Alkaline Washing

#### 3.2.1. Effect on Chlorides Removal and Calcium Retention

**Figure 4:**
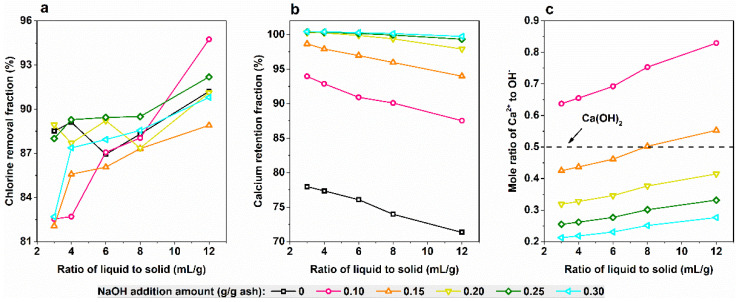
The fractions of chlorine removal (**a**) and calcium retention (**b**) after washing with various mole ratios of Ca^2+^/OH^−^ (**c**).

The MSWI fly ash was washed by alkaline solution with the NaOH addition amount ranging from 0.1 g/g ash to 0.3 g/g ash. The chlorine removal fraction and the calcium retention fraction after washing are shown in Figure 4a,b, respectively. As expected, the higher ratio of liquid to solid resulted in the higher chlorine removal fraction for more chlorides dissolved (Figure 4a). Unexpectedly, the NaOH addition amount had a great impact on chlorine removal (Figure 4a). When the ratio of liquid to solid was from 3 mL/g to 6 mL/g, the NaOH addition with the amount of 0.10, 0.15 and 0.30 g/g ash depressed the chlorides’ dissolution (same meaning as chlorine removal), resulting in the lower chlorine removal fraction. According to the common ion effect [27], more Na^+^ added into the washing slurry could reduce the solubility of NaCl. However, under the same ratio of liquid to solid, the NaOH addition with the amount of 0.20 and 0.25 g/g ash had no obvious effect on chlorine removal. The discontinuity of this trend suggests other effect or effects besides the common ion effect. The dissolution of CaClOH in ash was considered as its decomposition to soluble CaCl_2_ and slightly soluble Ca(OH)_2_. The addition of NaOH could react with soluble CaCl_2_ and produce slightly soluble Ca(OH)_2_, thus promoting the dissolution of CaClOH. Therefore, both effects of NaCl dissolution depression and CaClOH dissolution promotion existed simultaneously and caused the different effect of NaOH addition on the chlorine dissolution fraction. When the ratio of liquid to solid increased to higher than 8 mL/g, the effect of NaOH addition on chlorine removal became less, because the lower concentration of NaOH in slurry caused the weaker common ion effect.

In Figure 4b, the effect of alkaline washing on calcium retention was simple: that more NaOH addition and a higher ratio of liquid to solid resulted in higher calcium retention fraction. For water washing without NaOH addition, less than 80% calcium was retained in ash. For alkaline washing with NaOH addition, more than 90% calcium was retained in ash. When the NaOH addition amount was up to 2.0 g/g ash, almost all the calcium was retained. To analyze the availability of NaOH, the mole ratio of Ca^2+^ in a water washing filtrate added to OH^−^ in NaOH was calculated under various conditions and the results are shown in Figure 4c. The mole ratio higher than 0.5 (Equation (1)) means the insufficiency of NaOH for its reaction with Ca^2+^, while lower than 0.5 means the excess of NaOH. The washing condition with the mole ratio of Ca^2+^ to OH^−^ closest to 0.5 (Ca(OH)_2_) was L/S = 8 mL/g and 0.15 g NaOH / g ash, with the calcium retention fraction of 90%. The water washing without NaOH addition under the same L/S obtained the calcium retention fraction of 74%. This means the dissolved calcium was lowered from 26% to 10%, consequently corresponding to the NaOH availability of 62%. Therefore, the reuse of the filtrate is necessary to improve the economy of alkaline washing.

Considering the effect of chlorine removal and calcium retention as well as the operation cost, the condition of L/S = 6 mL/g and 0.20 g NaOH/g ash was chosen for ash upgrading, under which the chlorine removal fraction was 89% and the calcium retention fraction was nearly 100%. The washed ash accounted for 77% of the raw ash.

#### 3.2.2. Effect on Major Components

For the chlorine removal, the elements distribution in washed ash was greatly different from the raw ash. The main elements in raw ash (MSWI-FA) and upgraded ash by water washing (WW-MSMI-FA, L/S = 6 mL/g) and alkaline washing (AW-MSMI-FA, L/S = 6 mL/g and 0.20 g NaOH/g ash) are shown in Table 5. The removal of NaCl, KCl and CaClOH caused the decrease in chlorine, potassium and sodium and the increase in calcium, iron, silicon, aluminum, magnesium, et al. It is obvious that the alkaline washing has a better effect than the water washing on the chlorine removal and calcium retention.

**Figure 5:**
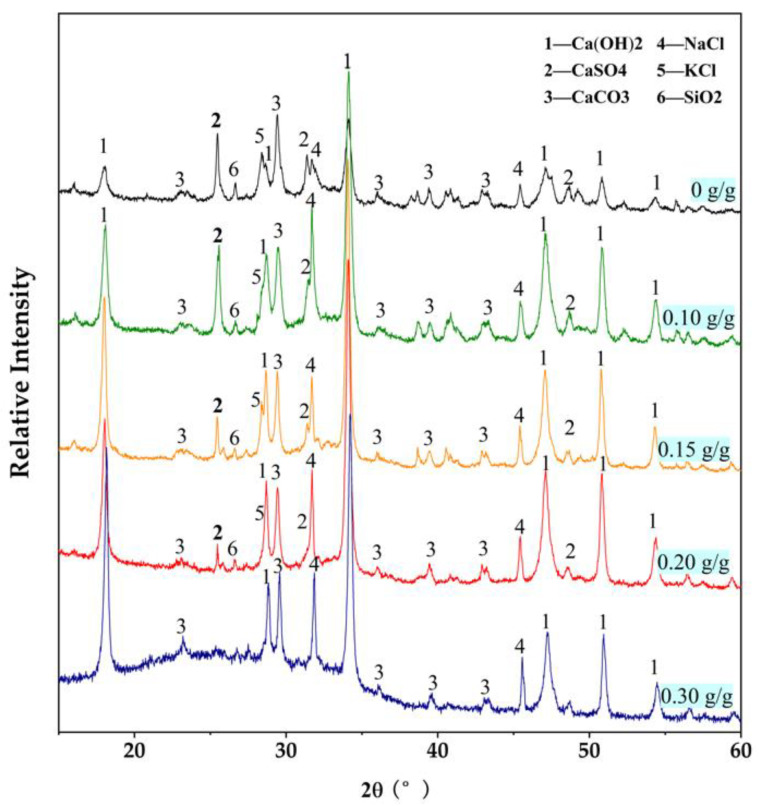
XRD patterns of ashes upgraded by water washing and alkaline washing (L/S = 6 mL/g).

As shown in Figure 2, the sulfur in MSWI fly ash mainly existed as CaSO_4_, which is slightly soluble in water. Puzzlingly, the alkaline washing decreased the content of sulfur in ash, indicating the possibility of CaSO_4_ dissolution in NaOH solution. In order to verify the dissolution of CaSO_4_ during alkaline washing, the washed ashes were analyzed in the crystal phase, as shown in Figure 5. Firstly, the increase in the peak intensities at 18.0°, 47.1° and 50.8° proved that calcium was retained in ash as Ca(OH)_2_. Secondly, the increase following decrease at 25.5° proved that CaSO_4_ was dissolved in NaOH solution. As shown in Figure 4c, theoretically, all the NaOH reacted with Ca^2+^ under the condition of L/S = 6 mL/g and 0.10 g NaOH / g ash, so that there was no residual NaOH left in the slurry and no CaSO_4_ dissolved, resulting in the decrease in the peak intensities at 25.5° under this washing condition. When the NaOH addition amount was up to 0.15 g/ g ash, NaOH was theoretically excessive for its reaction with Ca^2+^ at L/S = 6 mL/g, as shown in Figure 4c, so that part of CaSO_4_ was dissolved for NaOH in slurry. The improvement in CaSO_4_ dissolution by NaOH solution has been reported by Yuan et al. [28]. When the concentration of NaOH was higher or lower than 0.1 mol/L, CaSO_4_ was converted to soluble Ca(OH)^+^ and slightly soluble Ca(OH)_2_, respectively [28]. In addition, the conversion of CaSO_4_ to ettringite was found in the alkali activation process [29,30]. According to Figure 4c, the minimum amount of excessive NaOH was 0.15 g / g ash at L/S = 6 mL/g. Under this condition, the concentration of the rest of the NaOH in a washing slurry after reacting with Ca^2+^ was around 0.1 mol/L, theoretically. Thus, in all the conditions of NaOH excess, the concentration of NaOH should be higher than 0.1 mol/L, resulting in the conversion of CaSO_4_ to Ca(OH)_2_ and soluble Na_2_SO_4_, following Equation (5).

CaSO_4_ + 2 OH^−^ = Ca(OH)_2_ + SO_4_^2+^ (5)

#### 3.2.3. Heavy Metals Migration during Washing

**Figure 6:**
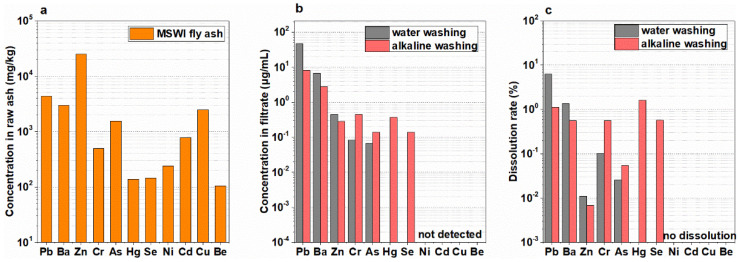
Heavy metals concentration in MSWI fly ash (**a**), filtrate (**b**) and their dissolution rate (**c**) after washing by water (L/S = 6 mL/g) and alkaline solution (L/S = 6 mL/g, 0.2 g NaOH/g ash).

Some speciation of heavy metals in MSWI fly ash were soluble, so they could migrate into the washing filtrate during washing, increasing the cost of wastewater treatment. The concentration of heavy metals in MSWI fly ash followed the sequence of Zn > Pb > Ba > Cu > As > Cd > Cr > Ni > Se > Hg > Be (Figure 6a), while the concentration of heavy metals in filtrate followed the sequence of Pb > Ba > Zn > Cr > As (Hg, Se, Ni, Cd and Cu were not detected) for water washing and the sequence of Pb > Ba > Cr > Hg > Zn > As and Se (Ni, Cd and Cu were not detected) for alkaline washing (Figure 6b). Only Pb and Ba had the concentration higher than 1 μg/mL in filtrate. Although Zn was the most abundant element in ash, its dissolution rate was lower than 0.1% (Figure 6c), so its concentration in filtrate was as low as about 0.3 μg/mL. Compared with water washing, alkaline washing depressed the dissolution of Pb, Ba and Zn, but improved that of Cr, As, Hg and Se. Considering that these depressed heavy metals had a high concentration in filtrate while these improved heavy metals had a low concentration, it is considered that alkaline washing depressed the migration of heavy metals from ash to filtrate, resulting in the cost decrease in wastewater treatment for heavy metals removal.

### 3.3. Performance of Prepared AACM Blocks

#### 3.3.1. Effect of Ashes on Setting Time of AACM Blocks

The setting time of AACMs prepared with MSWI fly ash or upgraded ash under different addition proportions are shown in Table 6. The great accelerating effects on the initial time and final time were found when MSWI fly ash or upgraded ashes were added. The significant reduction in setting time brought some negative effects when the MSWI fly ash addition amount was more than 20%. The initial setting time was too early to measure, and the final setting time was reduced to 23 s, indicating that more water and an alkali activator were needed. When the addition amount of water washing MSWI fly ash was more than 30%, the initial setting time could not be measured, and the final setting time was shortened to 49 s, which brought the same negative impact as MSWI fly ash. When the addition amount of alkaline washing MSWI fly ash was 10%, it showed the same procoagulant result as MSWI fly ash. However, when the addition amount increased to 20% and 30%, the initial and final setting times were obviously prolonged. The initial setting time of more than 3 min could meet the requirements of Binder Jetting 3D Printing [31].

**Figure 7:**
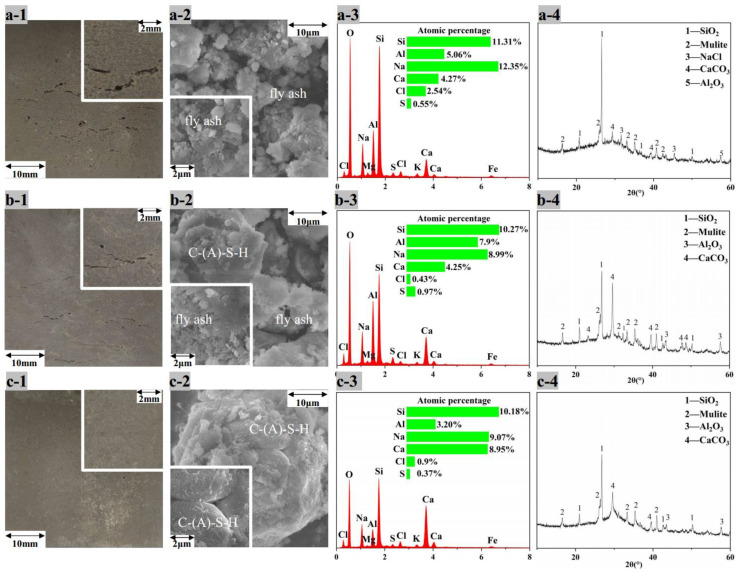
Appearance, micromorphology and elements distribution of surface and XRD patterns of AACM blocks (28 days) with 20% addition of (**a**) MSWI fly ash, (**b**) water washing ash and (**c**) alkaline washing ash.

Figure 7a-1, b-1 and c-1 show the appearance of AACM blocks with 20% of MSWI fly ash, water washing ash and alkaline washing ash, respectively, after standard molding and curing for 28 d. There were great cracks and expansion in Figure 7a-1, less cracks and expansion in Figure 7b-1 and no cracks and expansion in Figure 7c-1. For the creaked blocks, the compressive strength was too low to test. According to the results of Section 3.2.2, the speciation in MSWI fly ash was NaCl, KCl, CaClOH, CaSO_4_ and CaCO_3_; that in water washing ash were Ca(OH)_2_, CaSO_4_ and CaCO_3_; and that in alkaline washing ash was Ca(OH)_2_ and CaCO_3_. Therefore, the chlorides and the sulfate were considered to cause the cracking and expansion, and alkaline washing removed these chlorides and sulfate so that the blocks seemed better.

In order to analyze the reaction inside AACMs, the detections of the scanning electron microscope combined with energy dispersive spectroscopy (SEM-EDS) and XRD were preformed. The surface micromorphology in Figure 7a-2 shows the flocculent and fibrous aggregates of MSWI fly ash which seemed to be not involved in the reactions completely. In Figure 7b-2, the flocculent and fibrous aggregates were less and the particles larger than 10 μm were produced, probably for the formation of C-(A)-S-H [32,33]. In Figure 7c-2, the flocculent and fibrous aggregates disappeared and all the parts were connected. The EDS patterns show that washing pretreatment lowered the concentration of Na+ and Cl- in blocks. 

According to the XRD patterns in Figure 7, the peaks of SiO_2_ are the highest of the other crystals for all three AACM blocks. The residual SiO_2_ in the system without ash washing was more than that in the system with ash washing. The reaction between the CaClOH and NaOH (Equation (6)) [12] lowered the alkalinity in AACM, resulting in less SiO_2_ dissolution producing N-A-S-H. At the same time, the appearance of CaCl_2_ led to the instantaneous polymerization and gelation of silicate ions (Equation (9)) [34]. Moreover, the decomposition of CaClOH (Equation (7)) [35] in water produced CaCl_2_, which absorbed much water (Equation (8)), resulting in shortening the setting time and expanding the volume. Besides CaCO_3_, shown in Figure 7a-4, amorphous C-(A)-S-H gel could be another speciation of calcium. CaSO_4_ was not found in Figure 7a-4, and it could react to ettringite (AFt) and Friedel’s salt, which were expandable materials [36]. Alkaline washing converted both CaClOH and CaSO_4_ to produce Ca(OH)_2_, so the setting time was extended and the expansion and cracks became less. In addition, the slow release rate of Ca^2+^ for the low solubility of Ca(OH)_2_ lowered the reaction rate of Ca^2+^ with Na_2_O nSiO_2_ to form C-S-H, so the setting time was extended. The peaks of CaCO_3_ indicated its inertia, so it is infeasible to use Na_2_CO_3_ to retain calcium as CaCO_3_ during ash washing.

CaClOH + NaOH= Ca(OH)_2_ + NaCl (6)

2CaClOH = Ca(OH)_2_ + CaCl_2_ (7)

CaCl_2_ + 6H_2_O= CaCl_2_ 6H_2_O (8)

Na_2_O nSiO_2_ + CaCl_2_ = 2NaCl + CaO nSiO_2_↓ (9)

#### 3.3.2. Effect of Ashes on Compressive Strength of AACM Blocks

**Figure 8:**
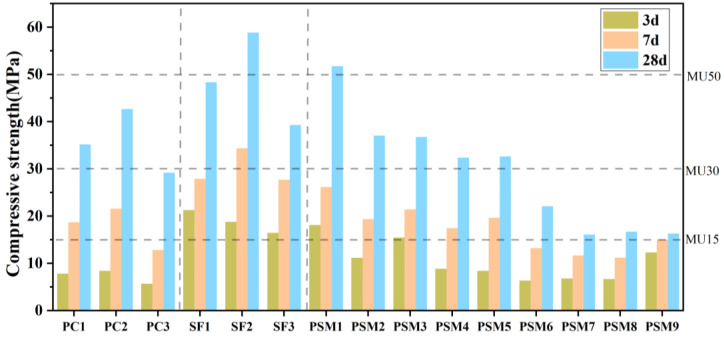
Compressive strength test results.

The compression strength of AACM blocks prepared following Table 2 and Table 3 are shown in Figure 8, and the longer curing time increased the strength greatly. The compressive strength of PC1, PC2 and PC3 indicated that the best modulus of alkali activator was 1.4 for preparing AACMs with pulverized coal combustion fly ash, and the maximum compressive strength was 42.65 MPa. A higher alkali activator modulus means less NaOH addition, resulting in the incomplete reaction of pulverized coal combustion fly ash dissolution and the decrease in compressive strength. A lower alkali activator modulus means more NaOH addition, resulting in more production of N-A-S-H which had high viscosity and fast setting and hardening. These N-A-S-H enclosed the rest of the unreacted particles, thus hindering the further reaction and lowering the compressive strength [37]. Next, the silica fume was added into the pulverized coal combustion fly ash with best modulus of 1.4, and the compressive strength of AACM blocks was marked as SF1, SF2 and SF3 in Figure 8. The maximum compressive strength of 58.83 MPa was corresponding to the addition proportion of 10% silica fume, not 5% or 15%. Wan et al. [38] found that the structure formed by N-A-S-H was determined by the Si/Al ratio; consequently, it had a great effect on mechanical strength. The best Si/Al ratio was considered as 2, corresponding to poly-sialate-siloxo [20].

The orthogonal test results of the compressive strength of AACM blocks are shown in Table 7. Within the selected factors and levels, the significance of the influencing factors for 3 d, 7 d and 28 d followed modulus of alkali activator > amount of silica fume > amount of AW-MSWI fly ash. According to the compressive strength of 28 d in Figure 8 (PSM 1–3), AW-MSWI fly ash reduced the compressive strength of AACM blocks when the alkali activator was sufficient. When the modulus was 1.4 (PSM 4–6), the decrease was 33-44.5% compared with the SF group and 24.1−48.2% compared with the PC group. The maximum compressive strength reached 32.61 MPa with 30% of AW-MSWI fly ash addition. When the modulus was 1.2 (PSM 1–3), the decrease was 4.4−47% compared with PC group. The compressive strength reached 51.72 MPa with 10% of AW-MSWI fly ash addition, and 36.73 MPa with 30% of AW-MSWI fly ash addition. When the modulus of alkali activator M = 1.6 (PSM 7–9), the compressive strength of AACMs prepared by adding 10%, 20% and 30% AW-MSWI fly ash was close, and the compressive strength of AACM blocks reached 16.06 MPa, meeting the MU15 grade in *Fired common bricks* (GB/T 5101-2017) [23]. A high modulus means less alkali activator. However, in these cases, with the modules of 1.6, the compressive strength was high enough. Therefore, Ca(OH)_2_ in AW-MSWI fly ash was considered to have the alkali activated effect like that of NaOH, to a certain extent. Generally, MU10 grade bricks can be used in the wall (above the moisture-proof course) of the building below the six stories, and MU30 grade bricks can be used in the wall of the buildings over six stories. Therefore, these prepared bricks with greater load-bearing capacity have a wide range of applications. In addition, the bricks made of fly ash have better thermal insulation and workability [39].

**Figure 9:**
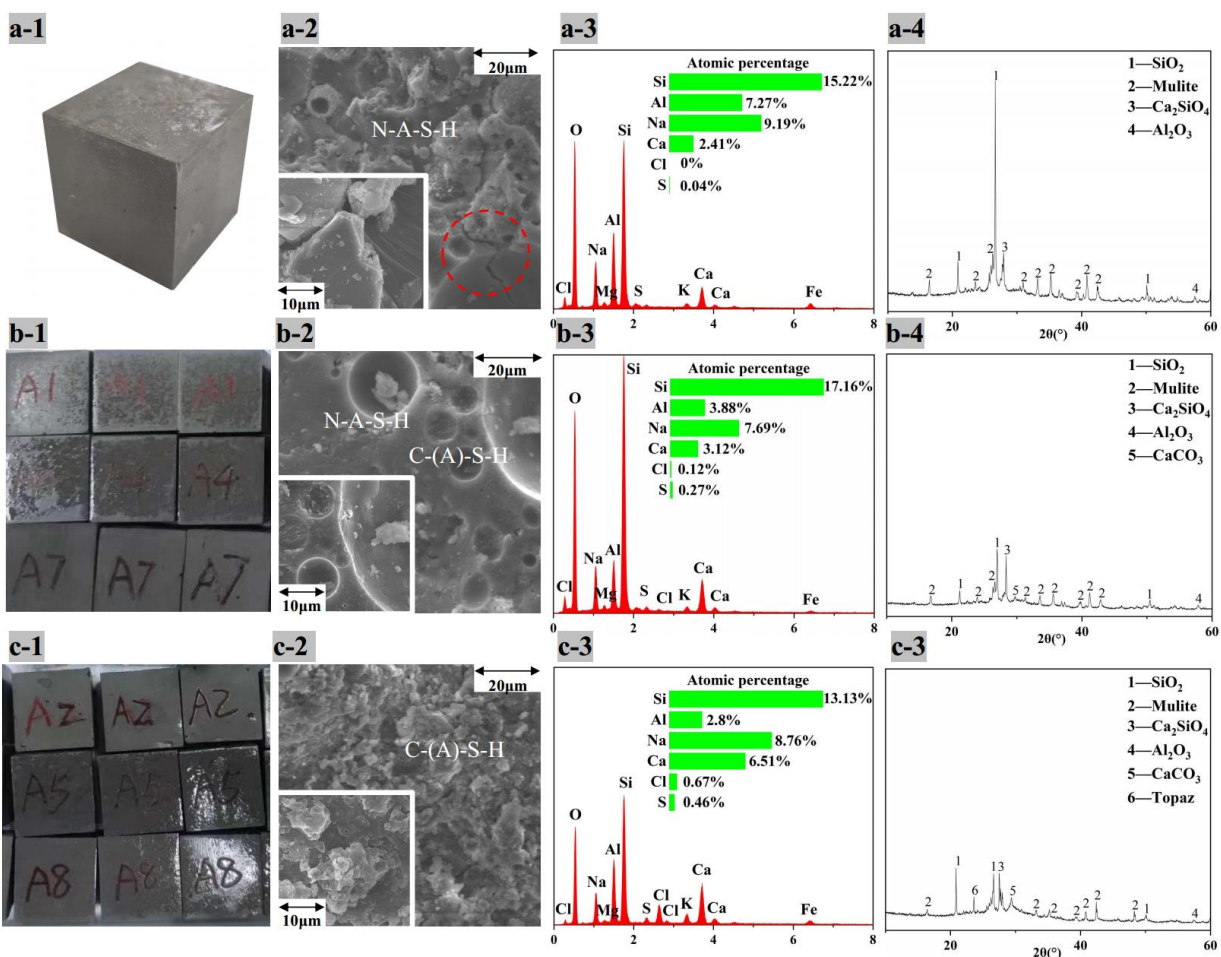
Appearance, micromorphology and elements distribution of surface and XRD patterns of AACM blocks (28 days). (**a**) PC1, (**b**) PSM1 and (**c**) PSM3.

Figure 9 shows the XRD, SEM-EDS results of 28 d AACM blocks prepared by pulverized coal combustion fly ash (PC1) and its mixture with alkaline washing MSWI fly ash and silica fume (PSM1 and PSM3). For PC1 block, the dense and continuous surface (Figure 9a-2) indicated the pulverized coal combustion fly ash was extensively converted to N-A-S-H gel, which was amorphous without diffraction peaks in Figure 9a-4 [40]. Although the density was relatively high, there were still small cracks, which were not enough to fill all the pores.

For the PSM1 block, the cracks on the gel surface were greatly reduced for the addition of 10% alkaline washing MSWI fly ash, as shown in Figure 9b-2. Compared with PC1 block, the PSM1 block had much lower peaks of SiO_2_ in the XRD pattern (Figure 9b-4). No crystalline phase of Ca was detected, indicating the conversion of Ca(OH)_2_ in upgraded fly ash to C-(A)-S-H gels which filled the cracks, wrapped and connecting the unreacted particles and finally promoting the compressive strength. When the proportion of alkaline washing MSWI fly ash was increased to 30%, the surface (Figure 9c-2) was no longer smooth and was without flocculent and fibrous particles, indicating more C-(A)-S-H gels formation.

#### 3.3.3. Effect on Heavy Metals Stabilization and Solidification

**Figure 10:**
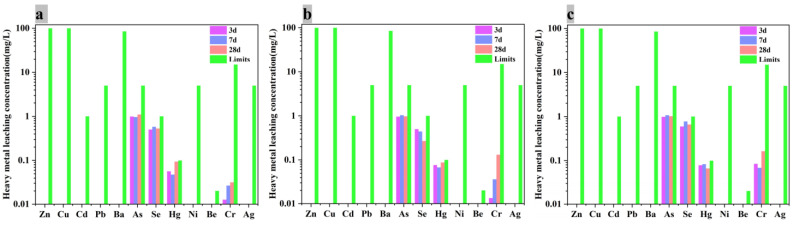
Heavy metal concentrations of leaching solutions. (**a**) PSM3, (**b**) PSM5 and (**c**) PSM7.

The AACM blocks with 30% of alkaline washing MSWI fly ashes (PSM3, PSM5 and PSM7) after curing for 3 d, 7 d and 28 d were leached following *Solid waste-Extraction procedure for leaching toxicity-sulphuric acid & nitric acid method* (HJ/T 299–2007) [41] to evaluate their fixation of heavy metals, and the results are shown in Figure 10 with the limits in *Identification standard of hazardous waste* (GB18598-2019) [42]. All the heavy metals detected did not exceed the standard limits, indicating that these blocks were not hazardous waste, at least, even with the 30% MSWI fly ash inside and the curing for 3 days. Encouragingly, there was almost no release during leaching for Zn, Pb, Cu and Ba, which have the highest concentrations in MSWI fly ash. In addition, Cd, Ni, Be, Cr and Ag were also fixed well. The physical wrapping by gels and the chemical embedding in the AACM’s skeleton to replace cations such as Na^+^, K^+^ or Ca^2+^ were both considered as the reason for the heavy metals fixation [12]. Although As, Se and Hg met the requirements for harmless treatment, their leaching concentrations were close to the limits. For the possible substandard situation, the addition proportion of alkaline washing MSWI fly ash should be less. In addition, the preparation of AACMs in this work did not include the mechanical grinding of the mixture of raw materials before alkaline activation. Therefore, the additional mechanical grinding could be in favor of the reactions of gels formation so that more heavy metals could be fixed effectively.

## 4. Conclusions

In this paper, MSWI fly ash was upgraded by low temperature calcination for dioxins removal and alkali washing for chlorides removal. The removal rate of dioxins reached 93% after calcination for 1 h at 450 °C under nitrogen atmosphere. Alkaline washing removed 89% of chlorine and retained almost 100% of calcium from MSWI fly ash with the addition of 0.2 g NaOH /g ash. In addition, excessive NaOH converted CaSO_4_ in ash to Ca(OH)_2_ and soluble Na_2_SO_4_. Compared with water washing, alkali washing inhibited the migration of most heavy metals from ash to filtrate, thus reducing the cost of wastewater treatment and leaving these heavy metals to AACMs for fixation. However, it promoted the dissolution of Hg and Se.

The initial setting time of AACMs was too short to detect for 20% of MSWI fly ash addition and the prepared block had extensive cracks and expansion for CaClOH and CaSO_4_ inside. Alkaline washing pretreatment increased the initial setting time by longer than 3 min with 30% ash addition for the conversion of CaClOH to Ca(OH)_2_. Besides, the cracks and the expansion were eliminated well for the formation of C-(A)-S-H and the conversion of CaSO_4_ to Ca(OH)_2_, respectively. The significance of the influencing factors for compressive strength followed the modulus of alkali activator > amount of silica fume > amount of AW-MSWI fly ash. When the activator modulus was 1.2, 1.4 and 1.6, the blocks with 30% of alkaline washing MSWI fly ash had a compressive strength up to 36.73 MPa, 32.61 MPa and 16.06 MPa, all meeting MU15 grade. The leaching test results show that these AACM blocks were not hazardous waste and almost no Zn, Cu, Cd, Pb, Ba, Ni, Be and Ag were released in the leaching solution.

## Figures and Tables

**Table 1:** Major elements of MSWI fly ash (MSWI-FA) and pulverized coal combustion fly ash (PCC-FA). Unit: %.

	CaO	Cl	SO_3_	K_2_O	Na_2_O	Fe_2_O_3_	SiO_2_	Al_2_O_3_	MgO	Other
MSWI-FA	45.7	22.5	9.7	6.7	7.2	1.3	2.4	1.0	0.9	2.2
PCC-FA	4.0	0	0.7	2.0	0.9	4.2	54.0	31.1	1.0	2.1

**Table 2:** Orthogonal test table.

Group	The Composition of AACM		
	Alkali Activator Modulus (A)	Silica Fume Addition/% (B)	AW-MSWI Fly Ash Addition/% (C)
PSM1	1.2	5	10
PSM2	1.2	10	20
PSM3	1.2	15	30
PSM4	1.4	5	20
PSM5	1.4	10	30
PSM6	1.4	15	10
PSM7	1.6	5	30
PSM8	1.6	10	10
PSM9	1.6	15	20

**Table 3:** Preparation parameters of AACMs with PCC-FA and SF.

Group	Alkali Activator Modulus	Composition
PC1	1.2	PCC-FA
PC2	1.4	
PC3	1.6	
SF1	1.4	PPC-FA:SF = 95:5
SF2		PPC-FA:SF = 80:10
SF3		PPC-FA:SF = 85:15

Note: PPC-FA represents pulverized coal combustion fly ash; SF represents silica fume.

**Table 4:** Dioxins distribution in MSWI fly ash and calcined ash.

Species		TEF	MSWI Fly Ash				Calcined Ash				Removal Fraction
			Concentration		TEQ		Concentration		TEQ		
			ng/kg	%	ng/kg	%	ng/kg	%	ng/kg	%	%
PCDFs	2,3,7,8- T4CDF	0.1	69.0	7.1	6.9	7.4	5.3	11.2	0.5	8.1	92.3
	1,2,3,7,8- P5CDF	0.05	89.0	9.1	4.5	4.8	5.3	11.2	0.3	4.1	94.0
	2,3,4,7,8- P5CDF	0.5	74.0	7.6	37.0	39.6	7.2	15.2	3.6	55.3	90.3
	1,2,3,4,7,8- H6CDF	0.1	56.0	5.7	5.6	6.0	3.9	8.2	0.4	6.0	93.0
	1,2,3,6,7,8- H6CDF	0.1	64.0	6.5	6.4	6.8	3.7	7.8	0.4	5.7	94.2
	2,3,4,6,7,8- H6CDF	0.1	46.0	4.7	4.6	4.9	3.9	8.2	0.4	6.0	91.5
	1,2,3,7,8,9- H6CDF	0.1	4.2	0.4	0.4	0.4	0.5	1.1	0.1	0.8	88.1
	1,2,3,4,6,7,8- H7CDF	0.01	110.0	11.3	1.1	1.2	6.7	14.2	0.1	1.0	93.9
	1,2,3,4,7,8,9- H7CDF	0.01	17.0	1.7	0.2	0.2	1.1	2.3	0.0	0.2	93.5
	O8CDF	0.001	33.0	3.4	0.0	0.0	1.2	2.5	0.0	0.0	96.4
PCDDs	2,3,7,8- T4CDD	1	9.3	1.0	9.3	9.9	0.0	0.0	0.0	0.2	99.9
	1,2,3,7,8- P5CDD	0.5	24.0	2.5	12.0	12.8	1.2	2.5	0.6	9.2	95.0
	1,2,3,4,7,8- H6CDD	0.1	11.0	1.1	1.1	1.2	0.9	1.9	0.1	1.4	91.8
	1,2,3,6,7,8- H6CDD	0.1	16.0	1.6	1.6	1.7	0.5	1.1	0.1	0.8	96.9
	1,2,3,7,8,9- H6CDD	0.1	15.0	1.5	1.5	1.6	0.6	1.3	0.1	0.9	96.0
	1,2,3,4,6,7,8- H7CDD	0.01	110.0	11.3	1.1	1.2	2.5	5.3	0.0	0.4	97.7
	O8CDD	0.001	230.0	23.5	0.2	0.2	2.8	5.9	0.0	0.0	98.8
∑(PCDFs + PCDDs)			977.5	100	93.5	100	47.32	100	6.5	100	93.0

**Table 5:** Main elements of MSWI fly ash and upgraded ash by water washing (L/S = 6 mL/g) and alkaline washing (L/S = 6 mL/g, 0.2 g NaOH/g ash), %.

	CaO	Cl	SO_3_	K_2_O	Na_2_O	Fe_2_O_3_	SiO_2_	Al_2_O_3_	MgO	Other
MSWI-FA	45.7	22.5	9.7	6.7	7.2	1.3	2.4	1.0	0.9	2.2
WW-MSMI-FA	52.8	5.7	19.2	1.7	2.1	1.7	7.6	1.8	3.2	4.2
AW-MSMI-FA	65.1	3.9	9.1	1.6	4.6	1.6	6.4	1.7	2.6	3.4

**Table 6:** Effect of fly ashes on setting time of AACMs.

Group	Fly Ash Dosage wt/%	Setting Time/s	
		Initial	Final
PCC-FA	/	4500	8700
MSWI-FA	10%	1110	3360
	20%	/	166
	30%	/	23s
WW-MSWI-FA	10%	1630	5040
	20%	254	646
	30%	/	49s
AW-MSWI-FA	10%	1026	2280
	20%	347	752
	30%	205	515

**Table 7:** Analysis of the range of test results by various factors.

Range	3 d Compressive Strength/MPa			7 d Compressive Strength/MPa			28 d Compressive Strength/MPa		
	A	B	C	A	B	C	A	B	C
K1j	44.71	33.70	31.11	66.92	55.27	50.53	125.47	100.13	90.50
K2j	23.57	26.21	32.27	50.28	50.19	51.77	87.05	86.33	85.66
K3j	25.71	34.09	30.61	37.83	49.56	52.73	49.04	75.09	85.40
k1j	14.90	11.23	10.37	22.31	18.42	16.84	41.82	33.38	30.17
k2j	7.86	8.74	10.76	16.76	16.73	17.26	29.02	28.78	28.55
k3j	8.57	11.36	10.20	12.61	16.52	17.58	16.35	25.03	28.47
Rj	6.33	2.63	0.55	9.69	1.90	0.73	25.47	8.35	1.70
Order	A > B > C			A > B > C			A > B > C		

Note: Kj is the sum of indexes (compressive strength) corresponding to each level of this factor; kj is the average of Kj; R is the range of this factor.

## Data Availability

The data presented in this study are available on request from the corresponding author.
